# Long-Noncoding RNA (lncRNA) in the Regulation of Hypoxia-Inducible Factor (HIF) in Cancer

**DOI:** 10.3390/ncrna6030027

**Published:** 2020-07-06

**Authors:** Dominik A. Barth, Felix Prinz, Julia Teppan, Katharina Jonas, Christiane Klec, Martin Pichler

**Affiliations:** 1 Research Unit of Non-Coding RNAs and Genome Editing in Cancer, Division of Clinical Oncology, Department of Internal Medicine, Comprehensive Cancer Center Graz; Medical University of Graz, 8036 Graz, Austria; dominik.barth@medunigraz.at (D.A.B.); felix.prinz@medunigraz.at (F.P.); julia.teppan@edu.uni-graz.at (J.T.); katharina.jonas@medunigraz.at (K.J.); christiane.klec@medunigraz.at (C.K.); 2 Department of Experimental Therapeutics, The University of Texas MD Anderson Cancer Center, Houston, TX 77030, USA

**Keywords:** lncRNA, long non-coding RNA, hypoxia, hypoxia-inducible factor, HIF, cancer

## Abstract

Hypoxia is dangerous for oxygen-dependent cells, therefore, physiological adaption to cellular hypoxic conditions is essential. The transcription factor hypoxia-inducible factor (HIF) is the main regulator of hypoxic metabolic adaption reducing oxygen consumption and is regulated by gradual von Hippel-Lindau (VHL)-dependent proteasomal degradation. Beyond physiology, hypoxia is frequently encountered within solid tumors and first drugs are in clinical trials to tackle this pathway in cancer. Besides hypoxia, cancer cells may promote HIF expression under normoxic conditions by altering various upstream regulators, cumulating in HIF upregulation and enhanced glycolysis and angiogenesis, altogether promoting tumor proliferation and progression. Therefore, understanding the underlying molecular mechanisms is crucial to discover potential future therapeutic targets to evolve cancer therapy. Long non-coding RNAs (lncRNA) are a class of non-protein coding RNA molecules with a length of over 200 nucleotides. They participate in cancer development and progression and might act as either oncogenic or tumor suppressive factors. Additionally, a growing body of evidence supports the role of lncRNAs in the hypoxic and normoxic regulation of HIF and its subunits HIF-1α and HIF-2α in cancer. This review provides a comprehensive update and overview of lncRNAs as regulators of HIFs expression and activation and discusses and highlights potential involved pathways.

## 1. Introduction

Hypoxic conditions are a challenge for oxygen-dependent mammalian cells requiring an adequate cellular response in order to adapt metabolic and proliferative processes. Hypoxia-inducible factor (HIF) is the main sensor of cellular oxygen levels as well as the main transcriptional regulator of cellular response to hypoxia [1]. There are several subunits of this protein, e.g., HIF-1α, HIF-2α and HIF-3α as well as the constitutively expressed HIF-1β, that become activated by dimerization of HIFα subunits with HIF-1β [1,2]. Physiologically, during normoxic conditions, HIF-1α and HIF-2α are constantly degraded through initial hydroxylation by prolyl hydroxylase (PHD) enzymes which further enables binding to the von Hippel-Lindau (VHL) protein [3,4]. Subsequently, this initiates ubiquitination of HIFα subunits by α-ketoglutarate-dependent dioxygenases thereby marking them for proteasomal degradation. However, under hypoxic conditions, PHDs are no longer active, consequently leading to HIFα accumulation and further dimerization with the HIF-1β subunit. This enables binding to hypoxia response elements (HRE) in the promoter regions of target genes, which aims to reduce cellular oxygen consumption by, for instance, activating anaerobic glycolysis or promoting angiogenesis [1,5]. As for HIF-3α, in contrast to HIF-1α and HIF-2α evidence suggests a negative regulatory influence on hypoxia-related gene expression, partially by competing for HIF-1β and acting as a transcription factor [6,7]. Overexpression of HIF-3α was related to attenuated angiogenesis and proliferation [8]. In cancer, hypoxic conditions within tumors are frequently encountered. Therefore, it is not surprising that an overexpression of HIFs can be observed across many cancer types. However, HIFs may also be upregulated in normoxia [9,10,11]. This significantly impacts tumor growth and progression as, for instance, HIF overexpression promotes cancer angiogenesis or activates glycolysis in addition to the aerobic metabolism compensating for the increased energy demands of fast proliferating cancer cells, which is known as the Warburg effect [12,13]. In addition, HIF activation is strongly associated with emerging drug resistance [14,15]. Thus, HIFs represent a powerful potential therapeutic target which is consequently being investigated in several clinical trials aiming to therapeutically target HIFs [16,17]. 

Apart from that, in the recent years, a growing body of evidence supports the involvement of non-coding RNAs including microRNAs (miRNAs) and long-noncoding RNAs (lncRNAs) in HIFs regulation in cancer [18,19,20]. 

**Figure 1:**
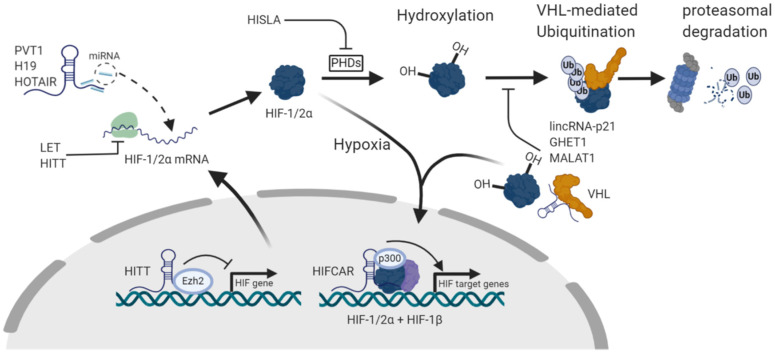
Regulation of hypoxia-inducible factor (HIF) and examples of long non-coding RNA (lncRNA) involvement. After transcription and translation under normoxic conditions hypoxia-inducible factor (HIF) 1/2α is hydroxylated by proline hydroxylases (PHD) which subsequently enables binding of the von Hippel-Lindau (VHL) protein and results in VHL-mediated ubiquitination and degradation through the proteasome pathway. In hypoxia, PHDs are inhibited and HIF-1/2α accumulate and are activated by dimerization with the constitutively expressed HIF-1β leading to transcription of HIF target gene. lncRNAs are involved in HIF regulation in several ways. For instance, they may regulate HIF transcription and translation, act as microRNA sponges, directly bind to PHDs and VHL or facilitate binding between HIF and transcriptional cofactors. (Created with BioRender.com).

lncRNAs are a class of non-protein coding RNAs that are more than 200 nucleotides in length [21]. In cancer, over the last decade lncRNAs were successfully established as regulators of tumor growth, progression and therapy resistance and are considered as potential therapeutic targets [22,23,24,25,26]. Additionally, their utility as diagnostic and prognostic biomarkers has been suggested [27]. LncRNAs can be classified depending on their localization within the genome. The sequences of intergenic lncRNAs (lincRNA) are localized between two protein-coding genes, whereas intronic lncRNAs originate from introns of protein-coding genes [28]. LncRNA transcripts may also overlap with known protein-coding genes. If they are transcribed in the opposite direction of a protein-coding DNA sequence, they are called antisense lncRNAs [28]. LncRNAs may exert their functions through different mechanisms, for example they may act as signal, guide, decoy or scaffold for other non-coding RNAs or proteins, thereby regulating processes like transcription, splicing, RNA stability and translation [29]. Several lncRNAs, such as HOXA distal transcript antisense RNA (HOTTIP) [30], prostate cancer gene expression marker 1 (PCGEM1) [31], gastric adenocarcinoma associated, positive CD44 regulator, long intergenic non-coding RNA (GAPLINC) [32] and antisense non-coding RNA in the INK4 locus (ANRIL) [33] are upregulated upon hypoxia, for instance, by binding of the HIF transcription factors to their promoter region. Yet not all of them are also exerting regulatory functions on the HIF pathway [34]. In the recent years, an increasing number of lncRNAs was reported to participate in HIFs regulation and acting directly or indirectly as enhancers or inhibitors of the HIF-pathway. Associated mechanisms include the regulation of HIFs transcription [35], translation [36], degradation [37], activation [38] and protein stability [39,40] (Table 1). See Figure 1 for an overview of HIF regulation and examples for lncRNA interventions.

The aim of this review is to summarize the current evidence on lncRNAs as regulators of HIFs across various cancer entities and to highlight so far unsolved questions that require further research. 

## 2. Methods

The PUBMED database was used for a literature search. The search terms used were “long non-coding”, “long non-coding”, “long noncoding”, “RNA”, “lncRNA”, “hypoxia”, “hypoxic”, “hypoxia-inducible factor”, “hypoxia inducible factor” or “HIF” in various combinations. Headlines and abstracts and full texts were screened for relevance. We excluded studies that solely addressed lncRNAs induced by HIF and only included studies that presented lncRNAs as direct or indirect regulators of HIF subsets.

## 3. lncRNAs in the Regulation of HIF-1α

### 3.1. lncRNAs as Enhancers of HIF-1α Expression, Activation and Stability

#### 3.1.1. PVT1

Plasmacytoma Variant Translocation 1 (PVT1) is a well-known oncogenic lncRNA and has been repeatedly suggested as a novel target in cancer therapy [70,71]. PVT1 may participate in the regulation of HIF-1α in two different ways. On the one hand, it was shown to act as a competing endogenous RNA (ceRNA) for the miRNAs miR-186 [43] and miR-199a-5p [42] in gastric cancer and non-small cell lung cancer (NSCLC) cells, respectively, thereby preventing them from miRNA-mediated HIF-1α mRNA degradation. Overexpression of PVT1 therefore resulted in increased HIF-1α expression levels [42,43]. On the other hand, a different mechanism of HIF-1α regulation was only recently proposed in nasopharyngeal carcinoma by Wang et al. [41]. PVT1 was shown to stabilize HIF-1α by acting as a scaffold for the chromatin modifying histone acetyltransferase KAT2A (lysine acetyltransferase 2A) thereby promoting its function and acetylation of H3K9. Consequently, this facilitates the binding of transcription intermediary factor 1β (TIF1β) and H3K9ac to the TIF1β/H3K9ac complex which then acts as a transcriptional activator for HIF-1α stabilizing genes. Wang et al. [41] identified increased NF90 (nuclear factor 90) transcription by overexpression of PVT1 and binding of the TIF1β/H3K9ac complex to the NF90 promoter region. NF90 is a double-stranded RNA-binding protein that ultimately stabilizes HIF-1α mRNA. This was experimentally confirmed both in vitro and in vivo when they also investigated the role of this pathway in radiotherapy sensitivity [41]. 

Apart from PVT1 [42,43], various other lncRNAs exert their regulatory function on HIF-1α by acting as ceRNAs [72,73,74,75,76,77,78,79,80,81,82,83,84,85,86,87,88,89,90]. See Table 2 for an overview of ceRNAs and their associated pathways in HIF regulation. Some selected examples with unique pathways or a larger body of evidence are further discussed in this section.

#### 3.1.2. H19

H19 is a well-known lncRNA described to play a role in several types of cancer [92,93]. Almost a decade ago Matouk et al. [94] showed that H19 is positively correlated with HIF-1α expression in hypoxic carcinoma cells. Overexpression of HIF-1α resulted in a significant increase of H19 levels, whereas suppression of HIF-1α led to the opposite result. Notably, these effects were observed in the absence of functional p53 tumor suppressor, indicating a regulatory mechanism between p53, HIF-1α and H19 in carcinogenesis [94]. This is in line with another study demonstrating HIF-1α-dependent expression of H19 by direct binding of HIF-1α to the H19 promoter. In addition, HIF-1α also induced transcription of specific protein 1 (SP1) which indirectly promoted H19 transcription [95]. Yet, more recent data implicate that not only HIF-1α is an upstream regulator of H19, but that, vice versa, H19 may also influence HIF-1α expression [88,89,90]. H19 may be involved in the regulation of HIF-1α through its ability to act as a ceRNA and by sponging multiple miRNAs involved in HIF-1α regulation. As shown in endometrial cancer cell lines, H19 is sponging miR-20b-5p which directly targets the 3′UTR of HIF-1α and thus inhibits HIF-1α expression [89]. Consequently, increased levels of H19 lead to elevated HIF-1α expression and subsequent activation of its downstream effectors [89]. In the study by Zhu et al. [89] H19 was found to promote endometrial cancer progression in both in vitro and in vivo experiments through the H19/HIF-1α/AXL pathway [89]. Another pathway involving H19 as a ceRNA was demonstrated by Peng and colleagues [88]. By binding miRNA let-7 higher expression of H19 in hypoxia promotes the release of HIF-1α in hypoxic breast cancer stem cells eventually resulting in elevated expression of pyruvate dehydrogenase kinase 1 (PDK1) and consequently enhanced glycolysis and stemness [88]. PDK1 has already been shown to be a direct target of HIF-1α and is an important factor in adapting mitochondrial function in hypoxia [96]. Interestingly, H19 features an embedded intragenic miRNA (miR-675-5p) which was demonstrated to directly induce hypoxic response through HIF-1α expression and activation under normoxic conditions in glioblastoma cells in vitro and in vivo [97]. Findings of Corrado et al. [90] eventually corroborate the previous studies by indicating an important involvement of H19 in HIF-1α regulation. They found that knockout of H19 impaired the nuclear translocation of HIF-1α in multiple myeloma cell lines under hypoxic conditions, thereby inhibiting its capability as a transcription factor. However, the knockdown of H19 was not found to result in a reduction of HIF-1α expression [90]. Collectively, the data discussed above imply a potential positive feedback loop between H19 and HIF-1α. However, this remains speculative since there are no current studies available which directly investigate on this matter. 

#### 3.1.3. HOTAIR

To date a growing body of evidence supports the role of lncRNA HOX transcript antisense intergenic RNA (HOTAIR) in cancer pathogenesis [98]. HOTAIR was reported to be upregulated in cancer cells under hypoxic conditions and involved in HIF-1α regulation by acting as a ceRNA [84,85,86,87]. Hu et al. [87] demonstrated increased HOTAIR expression in hepatocellular carcinoma which was increased even further under hypoxia. Via the HOTAIR/miR-130a-3p/HIF-1α axis, HOTAIR positively regulates HIF-1α expression resulting in enhanced glycolysis in hypoxic hepatocellular carcinoma cells. [87] As lncRNAs may act as decoys for multiple different miRNAs, HOTAIR also regulates HIF-1α expression by sponging the tumor suppressor miR-127, as first demonstrated in renal cell carcinoma (RCC) [84]. Additionally, upregulated HIF-1α resulted in a AXL receptor tyrosine kinase (AXL) expression, leading to enhanced proliferation, migration and EMT in RCC as demonstrated both in vitro and in vivo [84]. The proposed HOTAIR/miR-127/HIF-1α pathway was later confirmed in a study of Li et al. [86] which focused on HOTAIR’s impact on radioresistance in cervical cancer. Interestingly, the effect of the knockdown of HOTAIR could not be reversed by overexpression of HIF-1α indicating HIF-1α to be a definite downstream target [86]. However, these results conflict with previous data which reported HIF-1α mediated expression of HOTAIR in NSCLC cell lines upon hypoxia. HIF-1α was shown to directly bind to hypoxia-responsive elements of the HOTAIR promoter thus increasing HOTAIR expression [85]. Therefore, further research is required and may address the existence of a potential feedback-loop between HOTAIR and HIF-1α expression. 

#### 3.1.4. UCA1

Another lncRNA which is involved in a HIF-1α feedback-loop is urothelial carcinoma associated 1 (UCA1). In breast cancer cell lines, UCA1 upregulation is induced by tamoxifen treatment in a HIF-1α dependent manner [82]. Increased UCA1 expression by HIF-1α upregulation was also reported in osteosarcoma as well as hypoxic bladder cancer cells through HREs in the UCA1 promoter region [99,100]. Subsequently, miR-18a, which would otherwise directly target and therefore inhibit HIF-1α, is sponged by increasing UCA1 levels leading to a HIF-1α increase. This closes a positive feedback-loop and in addition promotes tamoxifen resistance in breast cancer cells [82]. 

#### 3.1.5. LINK-A

Long intergenic non-coding RNA for kinase activation (LINK-A) was found to regulate normoxic HIF-1α activation in triple negative breast cancer [46]. Briefly, the cytoplasmic lncRNA LINK-A is necessary for Heparin-binding EGF-like growth factor (HB-EGF)-mediated normoxic HIF-1α stabilization. Upon HB-EGF stimulation, LINK-A facilitates the recruitment of breast tumor kinase (BRK) by the epidermal growth factor receptor (EGFR)/transmembrane glycoprotein NMB (GPNMB) heterodimer complex to GPNMB resulting in the enzymatic activation of BRK. In addition, LINK-A also recruits and binds to leucine-rich repeat kinase 2 (LRRK2), which together with BRK phosphorylates HIF-1α at two specific sites, Tyr565 and Ser797, respectively. On the one hand, phosphorylation at Tyr565 limits the PHD protein-mediated hydroxylation of HIF-1α at Pro564, therefore inhibiting HIF-1α degradation under normoxia. On the other hand, Ser797 phosphorylation leads to the activation of the HIF-1α downstream signaling and transcription of target genes through facilitating the interaction between HIF-1α and p300 [46]. Interestingly, LINK-A expression was frequently increased in triple negative breast cancer tissue as compared to hormone receptor positive and HER2+ breast cancers and additionally was significantly associated with poor outcome [46]. Corroborating the findings of Lin et al. [46], a relationship of LINK-A and HIF-1α could also be observed in ovarian carcinoma as well as in osteosarcoma. In cell lines of both cancer types overexpression of LINK-A resulted in likewise increased HIF-1α levels and proliferation, migration and invasion [44,45]. The connection between LINK-A and HIF-1α was also reported in non-malignant diseases such as diabetic nephropathy, further supporting the existing body of evidence [101].

#### 3.1.6. lincRNA-p21

lincRNA-p21 may participate in HIF-1α regulation by enhancing HIF-1α stabilization [40]. This is achieved by competitive binding to the VHL protein which interferes with HIF-1α/VHL binding and consecutive ubiquitination and degradation of hydroxylated HIF-1α through the proteasome pathway [3,4,40]. Moreover, evidence suggests that HIF-1α and lincRNA-p21 may be connected in a positive feedback loop under hypoxic conditions. HIF-1α was demonstrated to promote lincRNA-p21 transcription by binding to hypoxia-related elements at the lincRNA-p21 promoter region. Yang et al. [40] also validated their results in in vivo experiments. The connection between lincRNA-p21 and HIF-1α in hypoxia was recently confirmed in liver cancer cells including xenograft models [102] as well as in a study investigating the influence of lincRNA-p21 on radio sensitivity of hypoxic cancer cells [103]. Interestingly, lincRNA-p21 may also regulate HIF-2α by direct binding to VHL as well [40].

#### 3.1.7. HISLA

HIF-1α expression may also be influenced via lncRNAs in the tumor microenvironment. Tumor associated macrophages (TAM) were repeatedly demonstrated to affect cancer progression by releasing cytokines and extracellular vesicles [104,105,106]. Interestingly, TAMs can also participate in HIF-1α regulation through the myeloid-specific lncRNA HIF-1α stabilizing long noncoding RNA (HISLA), as reported by Chen et al. [47]. Mechanistically, lactate emitted by glycolytic breast cancer cells induced HISLA upregulation in TAMs via ERK-ELK2 signaling, which was then released in the tumor microenvironment in extracellular vesicles and taken up by tumor cells. HISLA promotes HIF-1α stabilization by directly binding to prolyl hydroxylase domain 2 (PHD2), thereby forming a stem-loop formation. This interferes with PHD2-mediated HIF-1α hydroxylation and subsequent degradation, resulting in increased HIF-1α levels and enhanced chemoresistance as well as glycolysis in breast cancer. The latter may induce a feed-forward loop by leading to accumulation of lactate in the tumor microenvironment. This was demonstrated in both in vitro and in vivo experiments [47].

#### 3.1.8. GHET1

lncRNA gastric carcinoma high expressed transcript 1 (GHET1) may alter HIF-1α expression in two ways. On the one hand, GHET1 could activate the HIF-1α/Notch-1 signaling pathway via downregulating the tumor suppressor and transcription factor Kruppel-like factor 2 (KLF2) [48]. Zhu et al. [48] found that GHET1 is upregulated in prostate cancer cell lines and tissue and knockdown of GHET1 inhibits cancer cell proliferation and viability. In addition, the impact of GHET1 overexpression with consecutive inhibition of KLF2 and enhanced activation of HIF-1α/Notch-1 was demonstrated in a series of in vitro experiments [48]. Corroborating the results of Zhu et al. [48] KLF2 was previously reported to be connected to HIF-1α/Notch-1 activation [107] and GHET1 was already implicated in the regulation of KLF2 [108]. 

On the other hand, GHET1 may stabilize the HIF-1α protein and prevent it from degradation by VHL as demonstrated in in vitro experiments in ovarian cancer cell lines [39]. Mechanistically, GHET1 directly interacts with VHL preventing it from binding to HIF-1α [39]. Notably, both discussed mechanisms, GHET1/KLF2/HIF-1α/Notch-1 signaling and GHET1/VHL interaction, were investigated under normoxic conditions demonstrating the relevance of lncRNAs in HIF regulation apart from hypoxia [39,48]. 

#### 3.1.9. MIR31HG/HIFCAR

lncRNA miR-31 host gene (MIR31HG) is the host gene of miR-31 which is embedded in the first intron of the MIR31HG sequence [109]. MIR31HG is involved in cancer progression as reported in numerous studies [49,110,111,112,113,114]. After splicing and thus removal of the miRNA sequence, MIR31HG was demonstrated to act as a HIF-1α co-activator in oral cancer. Accordingly, the authors of the study named the mature lncRNA transcript HIF-1α co-activating RNA (HIFCAR) [38]. HIFCAR directly binds to HIF-1α thereby enabling enhanced binding of HIF-1α to its cofactor p300 which results in hypoxia-related gene transcription as demonstrated both in vitro and in vivo experiments. Since HIFCAR was also upregulated in oral cancer tissue and cells under normoxic conditions, it induced a pseudohypoxic state in cancer cells. Moreover, upregulated HIFCAR was found to represent an independent biomarker associated with reduced recurrence-free survival (RFS) (HR = 3.500, 95%CI 1.317–9.302, *p* = 0.012) and promotes development of metastases [38]. The role of MIR31HG/HIFCAR in the regulation of HIF-1α was confirmed in head and neck cancer cells where it also promoted cell proliferation and impacted apoptosis [49]. 

#### 3.1.10. DANCR

Differentiation antagonizing non-protein coding RNA (DANCR) was investigated in nasopharyngeal carcinoma, where it was found to be upregulated in cancer cell lines and tissue [50]. Moreover, a significant association of DANCR upregulation and poor overall survival (OS) (HR = 1.78, 95% CI 1.04–3.03, *p* = 0.034) and promotion of metastases in vitro and in vivo were reported [50]. This is due to DANCR’s ability to stabilize HIF-1α mRNA leading to enhanced HIF-1α expression. Mechanistically, DANCR was found to directly interact with double-strand RNA binding protein NF90 thus influencing the NF90/NF45 complex [50]. The NF90/NF45 complex is able to promote mRNA stability, as reported repeatedly [115,116]. NF90 was found to also stabilize HIF-1α mRNA [41].

#### 3.1.11. CASC9

Cancer susceptibility candidate 9 (CASC9) was shown to be significantly upregulated in both lung cancer and nasopharyngeal carcinoma cell lines and tissues [51,52]. Interestingly, in an RNA pull-down assay, CASC9 was demonstrated to directly bind to the HIF-1α protein in nasopharyngeal carcinoma, subsequently enhancing its stability [52]. Overexpression of CASC9 did not result in a likewise increase of HIF-1α mRNA levels, but increased transcription of HIF-1α target genes [52]. The stabilizing effect of CASC9 on HIF-1α was later confirmed in lung cancer by Jin et al. [51] who additionally proposed the existence of a positive feedback loop between HIF-1α and CASC9. Moreover, CASC9 overexpression was associated with proliferation and metastasis in lung cancer and enhanced glycolysis nasopharyngeal carcinoma, [51,52]. 

#### 3.1.12. MALAT1

Metastasis Associated Lung Adenocarcinoma Transcript 1 (MALAT1) is a highly abundant lncRNA in cancer and plays role in cancer development and progression [117]. Its involvement in HIF-1α stabilization was demonstrated by Luo et al. [53]. Arsenite-mediated upregulation of MALAT1 in arsenite-induced malignant transformation of hepatic L-02 cells resulted in dissociation of VHL from HIF-1α which subsequently inhibited HIF-1α ubiquitination and facilitated its accumulation. Furthermore, increased HIF-1α levels induced glycolysis [53]. Interestingly, results of other studies suggested a positive feedback loop between HIF-1α and MALAT1. Hu et al. [118] found MALAT1 to be upregulated in response to hypoxic conditions in lung adenocarcinoma cells, indicating that MALAT1 might be a hypoxia responsive lncRNA. Ikeda et al. [119] who showed that HIF-1α-mediated expression of the H3K9 demethylase lysine demethylase 3A (KDM3A) under hypoxic conditions caused an upregulation of MALAT1 in multiple myeloma. Reciprocally, MALAT1 then induced HIF-1α upregulation [119].

#### 3.1.13. MTA2TR

Upregulation of the lncRNA MTA2 transcriptional regulator RNA (MTA2TR) under hypoxic conditions was reported to enhance pancreatic cancer progression by increasing HIF-1α levels in a study by Zeng et al. [54]. Mechanistically, MTA2TR recruits the transcription factor activating transcription factor 3 (ATF3) to the promotor region of the metastasis associated 1 family member 2 (MTA2) protein and thereby enhances MTA2 expression [54]. MTA2′s association with HIF-1α was previously shown in pancreatic cancer, as MTA2 deacetylases HIF-1α and consequently increases HIF-1α stability [120]. In their study, Zeng et al. [54] could verify this relationship and found lncRNA MTA2TR as an upstream regulator of MTA2 and subsequentially HIF-1α expression and proposed a MTA2TR/ATF3/MTA2/HIF-1α asis. Moreover, they discovered an interesting positive feedback loop between MTA2TR and HIF-1α, as the latter acts as a transcriptional enhancer of MTA2TR expression [54]. 

#### 3.1.14. Other lncRNAs

There are several lncRNAs with a reported connection with HIF-1α but in fact no further data on the exact nature of the relationship (e.g., direct or indirect) or other involved regulating molecules have been described. Two of these lncRNAs are discussed in the following paragraph, noting that further research to clarify their role in HIF-1α regulation is needed. 

The lncRNA ubiquitin conjugating enzyme E2C pseudogene 3 (UBE2CP3) was found to promote the secretion of vascular endothelial growth factor (VEGF) in hepatocellular carcinoma, as demonstrated in a co-culture system [55]. Subsequent knockdown experiments revealed that this VEGF secretion is regulated through the activation of the ERK1/2/HIF-1α/VEGF pathway following frequent upregulation of UBE2CP3 in hepatocellular carcinoma cell. However, specific mechanisms of UBE2CP3 in the activation of the respective pathway, such as for instance binding sites and other effector molecules, were not described and are yet to be investigated [55]. 

The lncRNA associated with poor prognosis of hepatocellular carcinoma (AWPPH), also called MIR4435-2 host gene (MIR4435-2HG), is an oncogenic lncRNA which has been related to tumor proliferation and progression in a variety of cancer entities [121,122,123]. Zhang et al. [56] indicated a role in HIF-1α regulation in glioma cells, where overexpression of AWPPH came with likewise increase in HIF-1α levels. In addition, in their study AWPPH levels were successfully used to differentiate metastatic from non-metastatic glioma [56]. However, no further functional investigations on the connection between AWPPH and HIF-1α were conducted so far.

### 3.2. lncRNAs as Inhibitors of HIF-1α Expression, Activation and Stability

#### 3.2.1. LET

The impact of lncRNA DANCR on HIF stabilization by interacting with NF90 [50] was discussed in a previous paragraph. Interestingly, another lncRNA and its interaction with NF90/HIF-1α regulation was described even earlier [57]. The lncRNA low expression in tumor (LET) is frequently downregulated in cancer as already indicated by its name and is considered a tumor suppressor [124]. LET interacts with RNA binding protein NF90, however, in contrast to DANCR [50], it increases ubiquitination and subsequent degradation of NF90 [57,125]. Therefore, the stabilization of the HIF-1α mRNA through NF90 is diminished due to increased NF90 degradation. However, since LET is downregulated in malignant tissue, this inhibitory effect on HIF-1α is being omitted. Suppression of LET was shown to increase under hypoxic conditions which is mediated by the hypoxia-induced histone deacetylase 3 (HDAC3) resulting in decreased acetylation of histones H3 and H4 in the LET promoter region [57]. Interestingly, HDAC3 expression is directly increased by HIF-1α itself which indicates the existence of a positive feedback loop through the HIF-1α/HDAC3/lncRNA-LET/NF90 axis under hypoxia [57]. Moreover, an interesting interaction between the two HIF-1α regulating lncRNAs DANCR and LET was reported in gastric cancer. DANCR appears to be directly involved in epigenetic LET suppression through its association with EZH2 and HDAC3 [126]. This provides a second pathway by which DANCR promotes HIF-1α upregulation and highlights an interesting regulatory relationship between DANCR and LET, two lncRNAs with opposing effects on HIF-1α. 

#### 3.2.2. ENST00000480739

The lncRNA ENST00000480739 was first identified and investigated in pancreatic ductal adenocarcinoma, where it was is downregulated as compared to non-malignant tissue. ENST00000480739 additionally represents an independent biomarker for OS in pancreatic cancer patients receiving surgery (HR = 0.028, 95%CI 0.002–0.347, *p* = 0.005). Moreover, it was found to negatively regulate HIF-1α expression through transcriptional activation of osteosarcoma amplified 9 (OS-9) [58]. OS-9 has previously been shown to negatively impact HIF-1α expression by influencing its hydroxylation, VHL binding affinity, proteasomal degradation, and inhibition of HIF-1α target genes [58]. Therefore, the predominant downregulation of ENST00000480739 in pancreatic cancer favors HIF-1α activation and promotes pancreatic cancer invasion as demonstrated in vitro and in vivo. Upregulation of ENST00000480739 might represent a promising future therapeutic target in cancer treatment [58].

#### 3.2.3. CPS1-IT1

Tumor suppressor lncRNA CPS 1 intronic transcript 1 (CPS1-IT1) may inhibit HIF-1α activation by binding to the chaperone heat shock protein 90 (Hsp90) which interferes with Hsp90′s binding affinity to HIF-1α [61]. As CPS1-IT1 expression is significantly decreased in hepatocellular carcinoma tissue and cell lines this results in promotion of epithelial-mesenchymal transition (EMT) and increased metastatic potential in vivo through HIF-1α activation. Additionally, reduced CPS1-IT1 levels represent an independent biomarker for disease-free survival (DFS) (HR = 0.55, 95%CI 0.34–0.87, *p* = 0.011) and OS (HR = 0.57, 95%CI 0.34–0.98, *p* = 0.042) in hepatocellular carcinoma patients [61]. A further study by Wang et al. [60] revealed that melatonin acts as upstream regulator of CPS1-IT1 through increased forkhead box A2 (FOXA2) expression in hepatocellular carcinoma cells, proposing a melatonin/FOXA2/CPS-IT1/HIF-1α pathway. In addition, reduced CPS1-IT1 expression and its ability of HIF-1α regulation was observed in colorectal cancer [59], corroborating the results of Wang et al. [60,61]. 

#### 3.2.4. HITT

In 2019, lncRNA HIF-1α inhibitor at translation level (HITT) was first described, with its name already defining one of its key functions. HITT is closely connected to HIF-1α as shown by two recent studies [35,36]. First, HITT interferes with the translation of HIF-1α by acting as a decoy for the Y box binding protein 1 (YB-1) protein, which represents a translational regulator of HIF-1α [36]. Consequently, frequent downregulation of HITT in cancer results in increased HIF-1α expression. Moreover, forming a regulatory feedback loop, HIF-1α induces miR-205 expression, which directly targets HITT resulting in its degradation and suppression, indicating HITT suppression is a necessary step for cellular hypoxic response [36]. Second, HITT interacts with polycomb repressive complex 2 (PRC2) core protein EZH2, which conducts chromatin silencing together with its substrate lysine 27 of histone 3 (H3k27) [36,127]. HITT recruits EZH2 to the promoter of the HIF-1α gene where it forms a triplex with the promoter sequence resulting in decreased HIF-1α transcription [35]. Therefore, one the one hand HITT may regulate HIF-1α translation [36], on the other hand it may as well influence its transcriptional activity [35].

#### 3.2.5. MEG3

Interesting findings by Zhou et al. [62] suggest a role of the lncRNCA maternally expressed gene 3 (MEG3) in the malignant transformation of bronchial epithelial cells driven by nickel exposure by affecting HIF-1α expression. Mechanistically, upon nickel exposure, the authors recognized downregulation of MEG3 by hypermethylation through increased expression of DNA methyltransferase 3 beta (DNMT3b). This resulted in subsequent PH domain and leucine rich repeat protein phosphatase 1 (PHLPP1) transcription inhibition, as the inhibitory effect of MEG3 on transcription factor c-Jun was reduced following MEG3 suppression [62]. PHLPP1 is a known inhibitor of the Akt pathway [128]. Consequently, nickel induced MEG3 downregulation eventually led to activation of the Akt/p70S6K/S6/HIF-1α pathway, increasing HIF-1α expression as demonstrated by Zhou et al. [62].

#### 3.2.6. IDH1-AS1

Another HIF-1α-suppressing lncRNA was identified by Xiang and coworkers [37], who found that c-Myc mediated suppression of lncRNA isocitrate dehydrogenase 1 antisense RNA 1 (IDH1-AS1) in cancer cell lines activates HIF-1α-induced glycolysis under normoxic conditions. A role of c-Myc in the upregulation of glycolysis in normoxic cancer cells was previously reported [129]. Mechanistically, IDH1-AS1 seems to promote IDH1 enzymatic activity by homo-dimerization [37]. Subsequently, induction of α-ketoglutarate, an electron donor of PHD in HIF-1α hydroxylation and degradation [130], and decrease of ROS production suppress HIF-1α [37]. Thus, upon IDH1-AS1 inhibition by c-Myc, the inhibitory effect on HIF-1α ceases to apply, resulting in HIF-1α upregulation and activation. This was also demonstrated in xenograft models [37].

#### 3.2.7. PIN1-v2

Interestingly, non-coding variants of otherwise protein-coding RNA sequences may also exert inhibitory function on HIF-1α expression at the transcriptional level [63]. For instance, the enzyme peptidyl-prolyl cis-trans isomerase NIMA-interacting 1 (PIN1), which itself was suggested to regulate HIF-1α activity and expression via different proposed functions [131,132,133], also has three non-protein coding variants which are considered lncRNAs. Among these, PIN1-v2 is able to inhibit HIF-1α expression at the transcriptional level via the transcription factor NFAT, as demonstrated by Choi et al. [63]. However, in their study, upregulation of the protein PIN1 alone had no effect on HIF-1α levels in contrast to previous studies [131,132,133]. 

#### 3.2.8. HOTAIRM1

The spliced HM1-3 isoform of the lncRNA HOXA transcript antisense RNA, myeloid specific 1 (HOTAIRM1) was reported to post-transcriptionally inhibit HIF-1α expression [64]. HM1-3 was demonstrated to be downregulated in clear cell renal cell carcinoma and upregulation could successfully inhibit HIF-1α expression in normoxic cells. However, a detailed analysis to elucidate the exact mechanisms of HOTAIRM1 and HIF-1α regulation is yet to be performed [64].

## 4. lncRNA and Regulation of HIF-2α

### 4.1. HIF2PUT

Hypoxia-inducible factor-2α promoter upstream transcript (HIF2PUT) is a lncRNA which is located on chromosome 2p21 on the antisense side of the promoter upstream region of the HIF-2α gene. It was first described in 2015 by Wang et al. [67] who found that HIF2PUT positively correlates with HIF-2α expression levels in osteosarcoma tissue and cell lines (R = 0.589, *p* = 0.013), and that overexpression and knockout of HIF2PUT could enhance or suppress HIF-2α mRNA levels, respectively. These results were later confirmed in colorectal cancer where HIF2PUT overexpression was related to stem cell-like properties [66]. Recently, HIF2PUT expression and its relationship to HIF-2α was again investigated in osteosarcoma stem cells. In this study—contrary to the results in colorectal cancer [66]—the overexpression of the respective lncRNA resulted in the inhibition of osteosarcoma stem cell proliferation, migration and invasion indicating its role as a tumor suppressor in osteosarcoma [65]. Also in this study the authors observed a positive relation between HIF2PUT and HIF-2α expression levels, corroborating the results of previous studies [65,66,67]. In addition, another study proposed the clinical utility of HIF2PUT as a biomarker in, as HIF2PUT overexpression was significantly and independently associated with shorter OS (HR = 5.476, 95%CI 1.993–12.286, *p* = 0.01) and DFS (HR = 5.936, 95%CI 1.312–12.688, *p* = 0.01) [134]. Even so, this contradicts the previously discussed findings of HIF2PUT being a potential tumor suppressor in osteosarcoma [65,67]. Furthermore, none of the aforementioned studies elucidated the specific mechanisms or pathways that underlie the regulation of HIF-2α by HIF2PUT. Therefore, these results may be considered as first steps towards unravelling the influence of HIF2PUT on HIF-2α and further research is required to give insight into how HIF2PUT’s downstream signaling regulates HIF-2α.

### 4.2. SARCC

Zhai et al. [68] were first to identify another lncRNA in the regulation of HIF-2α, which they named suppressing androgen receptor in renal cell carcinoma (SARCC). SARCC influences HIF-2α expression in a VHL-dependent manner, indicating different responses to hypoxia in VHL wildtype and VHL mutant RCC patients. Mechanistically, SARCC can directly bind to the androgen receptor (AR) protein leading to its enhanced ubiquitination and thus enhanced degradation. As a result, HIF-2α, c-MYC and its further downstream effectors were inhibited [68]. Posttranscriptional influence of AR on HIF-2α expression had already been demonstrated previously [135,136], and was further complemented by the findings of Zhai et al. [68] who indicate that AR may also directly interfere with HIF-2α transcription. Interestingly, HIF-2α itself may bind to HREs in the SARCC promoter region leading to suppression of SARCC in HIF-2α overexpression. In summary, considering the proposed VHL-dependent SARCC/AR/HIF-2α/c-MYC axis, VHL-wildtype RCC may experience a proliferation reduction under hypoxic conditions because of SARCC upregulation, whereas SARCC downregulation under hypoxia in VHL mutant RCC could lead to enhanced tumor proliferation [68]. 

### 4.3. MALAT1

MALAT1 is not only involved in the regulation of HIF-1α [53] (as discussed earlier), but it also plays a role in HIF-2α modulation [69]. In arsenite-mediated tumor development in hepatic epithelial cells, MALAT1 promotes HIF-2α stabilization by enhancing its dissociation from VHL. This results in HIF-2α accumulation, which is mechanistically, similar to its role in HIF-1α regulation [53,69]. Likewise, a positive feedback mechanism was proposed with HIF-2α regulating the transcriptional activity of MALAT1 [69]. This is in line with another study demonstrating HIF-2α mediated expression of MALAT1 in hepatocellular carcinoma cells [137]. 

### 4.4. NEAT1

Nuclear-enriched abundant transcript 1 (NEAT1) is an important lncRNA with known influence on carcinogenesis and tumor progression in various cancer entities [138,139]. Moreover, its role as a hypoxia responsive lncRNA transcriptionally induced by HIF-2α has been evaluated in a variety of studies, demonstrating its influence on invasion, metastasis or apoptosis [140,141,142,143]. Vice versa, NEAT1 may also regulate HIF-2α by acting as a ceRNA and sponging miR-186-5p, which directly targets HIF-2α. Therefore, upregulation of NEAT1 comes with decreased miR-186-5p and increased HIF-2α levels as shown in osteosarcoma cell lines [91]. This could indicate a feedback loop and regulatory relationship between HIF-2α and NEAT1. 

### 4.5. lincRNA-p21

As already discussed earlier, lincRNA-p21 may stabilize HIF-1α and preventing it from ubiquitination and degradation by competitively binding to VHL [40]. The same mechanism also results in the stabilization of HIF-2α [40].

## 5. Conclusions

In this review, the broad influence of lncRNAs on HIF-1α and HIF-2α expression, stability and activation as well as on its further downstream signaling has been summarized. lncRNAs may function as direct or indirect regulators of HIFs in cancer and are able to enhance or inhibit its function through diverse mechanisms under both normoxic and hypoxic conditions. Furthermore, regulatory feedback loops between HIFs and several lncRNAs may exist. However, we could not find any related studies that demonstrated lncRNAs in the regulation of HIF-3α, specifically. Thus, this remains the subject of further investigations. 

In conclusion, since the activation of the HIF-pathway in cancer changes the metabolic state towards glycolysis in addition to aerobic metabolism and promotes proliferation, angiogenesis and drug resistance in cancer cells [12,13,14,15], lncRNAs could represent promising therapeutic targets to influence HIF signaling in both hypoxic and normoxic conditions in human cancer. 

## Figures and Tables

**Table 1:** Overview of long non-coding RNAs involved in HIFs regulation, not including competing endogenous RNAs.

	lncRNA	Impact on HIF	Role in HIF Regulation	References
**HIF-1α**				
	PVT1	↑	increases HIF-1α expression and stability	[41,42,43]
	LINK-A	↑	increases HIF-1α stability and activation	[44,45,46]
	lincRNA-p21	↑	increases HIF-1α protein stability	[40]
	HISLA	↑	increases HIF-1α protein stability	[47]
	GHET1	↑	increases HIF-1α expression and stability	[39,48]
	MIR31HG/HIFCAR	↑	enhances HIF-1α activation	[38,49]
	DANCR	↑	stabilizes HIF-1α mRNA	[50]
	CASC9	↑	increases HIF-1α protein stability	[51,52]
	MALAT1	↑	increases HIF-1α protein stability	[53]
	MTA2TR	↑	increases HIF-1α protein stability	[54]
	UBE2CP3	↑	no specific mechanism defined	[55]
	AWPPH	↑	no specific mechanism defined	[56]
	LET	↓	decreases HIF-1α mRNA stability	[57]
	ENST00000480739	↓	decreases HIF-1α protein stability	[58]
	CPS1-IT1	↓	decreases HIF-1α activation	[59,60,61]
	HITT	↓	inhibits HIF-1α transcription and translation	[35,36]
	MEG3	↓	Increases HIF-1α expression	[62]
	IDH1-AS1	↓	decreases HIF-1α protein stability	[37]
	PIN1-v2	↓	inhibits HIF-1α transcription	[63]
	HOTAIRM1	↓	post-transcriptionally inhibits HIF-1α expression	[64]
**HIF-2α**				
	HIF2PUT	↑	no specific mechanism defined, increases expression	[65,66,67]
	SARCC	↓	decreases HIF-2α transcription and translation	[68]
	MALAT1	↑	increases HIF-2α protein stability	[69]
	lincRNA-p21	↑	increases HIF-2α protein stability	[40]

Abbreviations: PVT1—Plasmacytoma Variant Translocation 1; LINK-A—Long intergenic non-coding RNA for kinase activation; HISLA—HIF-1α stabilizing long noncoding RNA; GHET1—gastric carcinoma high expressed transcript 1; MIR31HG—miR-31 host gene; HIFCAR—HIF-1α co-activating RNA; DANCR—differentiation antagonizing non-protein coding RNA; CASC9—cancer susceptibility candidate 9; MALAT1—metastasis associated lung adenocarcinoma transcript 1; MTA2TR—MTA2 transcriptional regulator RNA; UBE2CP3—ubiquitin conjugating enzyme E2C pseudogene 3; AWPPH—associated with poor prognosis of hepatocellular carcinoma; LET—low expression in tumor; CPS1-IT1—CPS 1 intronic transcript 1; HITT—HIF-1α inhibitor at translation level; MEG3—maternally expressed gene 3; IDH1-AS1—isocitrate dehydrogenase 1 antisense RNA 1; PIN1-v2—enzyme peptidyl-prolyl cis-trans isomerase NIMA-interacting 1 variant 2; HOTAIRM1—HOXA transcript antisense RNA, myeloid specific 1; HIF2PUT—Hypoxia-inducible factor-2α promoter upstream transcript; SARCC—suppressing androgen receptor in renal cell carcinoma.

**Table 2:** Competing endogenous RNAs in the regulation of hypoxia-inducible factor.

	lncRNA	Cancer	Expression Pattern	Impact on HIF Expression	Pathway	Reference
**HIF-1α**						
	PVT1	Gastric cancer	↑	increase	PVT1/miR-186/HIF-1α	[43]
		Non-small cell lung cancer	↑	increase	PVT1/miR-199a-5p/HIF-1α	[42]
	H19	Endometrial cancer	↑	increase	H19/miR-20b-5p/HIF-1α/AXL	[89]
		Breast cancer	↑	increase	H19/miR-let-7/HIF-1α/PDK1	[88]
	HOTAIR	Hepatic cancer	↑	increase	HOTAIR/miR-130a-3p/HIF-1α	[87]
		Renal cell carcinoma	↑	increase	HOTAIR/miR-217/HIF-1α/AXL	[84]
		Cervical cancer	↑	increase	HOTAIR/miR-127/HIF-1α	[86]
	HIF1A-AS2	Breast cancer	↑	increase	HIF1A-AS2/miR-548c-3p/HIF-1α/VEGF	[83]
	UCA1	Breast cancer	↑	increase	UCA1/miR-18a/HIF-1α	[82]
	CDKN2B-AS1	Ovarian cancer	↑	increase	CDKN2B-AS1/miR-411-3p/HIF-1α/VEGF	[81]
	DLX6-AS1	Nasopharyngeal carcinoma	↑	increase	DLX6-AS1/miR-199a-5p/HIF-1α	[80]
	FEZF1-AS1	Pancreatic cancer	↑	increase	FEZF1-AS1/miR-142/HIF-1α	[79]
	LINC00512	Gallbladder carcinoma	↑	increase	LINC00512/miR-138/HIF-1α	[78]
	RoR	Hepatic cancer	↑	increase	RoR/miR-145/HIF-1α	[77]
	SNHG6	Esophageal squamous cell carcinoma	↑	increase	SNHG6/miR-186-5p/HIF-1α	[76]
	TMPO-AS1	Retinoblastoma	↑	increase	TMPO-AS1/miR-199a-5p/HIF-1α	[75]
	TUG1	Osteosarcoma	↑	increase	TUG1/miR-143-5p/HIF-1α	[74]
	XIST	Colorectal cancer	↑	increase	XIST/miR-93-5p/HIF-1α/AXL	[73]
	ZEB2-AS1	Gastric cancer	↑	increase	ZEB2-AS1/miR-143-5p	[72]
**HIF-2α**						
	NEAT1	Osteosarcoma	↑	increase	NEAT1/miR-186-5p/HIF-2α	[91]

Abbreviations: HOTAIR—HOX transcript antisense intergenic RNA, HIF1A-AS2—hypoxia-inducible factor-1 alpha antisense RNA-2, UCA1—urothelial carcinoma associated 1, CDKN2B-AS1—cyclin dependent kinase inhibitor 2B antisense RNA 1, DLX6-AS1—distal-less homeobox 6 antisense RNA 1, FEZF1-AS1—FEZ family zinc finger 1 antisense RNA 1, RoR—regulator of reprogramming, SNHG6—small nucleolar RNA host gene 6, TMPO-AS1—Thymopoietin antisense RNA 1, TUG1—taurine up-regulated 1, XIST—X inactive specific transcript, ZEB2-AS1—zinc finger E-box binding homeobox 2 antisense RNA 1, NEAT1—nuclear paraspeckle assembly transcript.

## References

[1] Schofield C.J., Ratcliffe P.J. (2004). Oxygen Sensing by HIF Hydroxylases. Nat. Rev. Mol. Cell Biol..

[2] Loboda A., Jozkowicz A., Dulak J. (2010). HIF-1 and HIF-2 Transcription Factors--Similar but Not Identical. Mol. Cells.

[3] Kaelin W.G., Ratcliffe P.J. (2008). Oxygen Sensing by Metazoans: The Central Role of the HIF Hydroxylase Pathway. Mol. Cell.

[4] Zhang J., Zhang Q. (2018). VHL and Hypoxia Signaling: Beyond HIF in Cancer. Biomedicines.

[5] Semenza G.L. (2007). Hypoxia-Inducible Factor 1 (HIF-1) Pathway. Sci. STKE.

[6] Yang S.L., Wu C., Xiong Z.F., Fang X. (2015). Progress on Hypoxia-Inducible Factor-3: Its Structure, Gene Regulation and Biological Function (Review). Mol. Med. Rep..

[7] Duan C. (2016). Hypoxia-Inducible Factor 3 Biology: Complexities and Emerging Themes. Am. J. Physiol. Cell. Physiol..

[8] Ando H., Natsume A., Iwami K., Ohka F., Kuchimaru T., Kizaka-Kondoh S., Ito K., Saito K., Sugita S., Hoshino T. (2013). A Hypoxia-Inducible Factor (HIF)-3α Splicing Variant, HIF-3α4 Impairs Angiogenesis in Hypervascular Malignant Meningiomas with Epigenetically Silenced HIF-3α4. Biochem. Biophys. Res. Commun..

[9] Jing X., Yang F., Shao C., Wei K., Xie M., Shen H., Shu Y. (2019). Role of Hypoxia in Cancer Therapy by Regulating the Tumor Microenvironment. Mol. Cancer.

[10] Manoochehri Khoshinani H., Afshar S., Najafi R. (2016). Hypoxia: A Double-Edged Sword in Cancer Therapy. Cancer Investig..

[11] Courtnay R., Ngo D.C., Malik N., Ververis K., Tortorella S.M., Karagiannis T.C. (2015). Cancer Metabolism and the Warburg Effect: The Role of HIF-1 and PI3K. Mol. Biol. Rep..

[12] Nagao A., Kobayashi M., Koyasu S., Chow C.C.T., Harada H. (2019). HIF-1-Dependent Reprogramming of Glucose Metabolic Pathway of Cancer Cells and its Therapeutic Significance. Int. J. Mol. Sci..

[13] Vander Heiden M.G., Cantley L.C., Thompson C.B. (2009). Understanding the Warburg Effect: The Metabolic Requirements of Cell Proliferation. Science.

[14] Ai Z., Lu Y., Qiu S., Fan Z. (2016). Overcoming Cisplatin Resistance of Ovarian Cancer Cells by Targeting HIF-1-Regulated Cancer Metabolism. Cancer Lett..

[15] Xu K., Zhan Y., Yuan Z., Qiu Y., Wang H., Fan G., Wang J., Li W., Cao Y., Shen X. (2019). Hypoxia Induces Drug Resistance in Colorectal Cancer through the HIF-1alpha/miR-338-5p/IL-6 Feedback Loop. Mol. Ther..

[16] Fallah J., Rini B.I. (2019). HIF Inhibitors: Status of Current Clinical Development. Curr. Oncol. Rep..

[17] Chen W., Hill H., Christie A., Kim M.S., Holloman E., Pavia-Jimenez A., Homayoun F., Ma Y., Patel N., Yell P. (2016). Targeting Renal Cell Carcinoma with a HIF-2 Antagonist. Nature.

[18] Choudhry H., Harris A.L. (2018). Advances in Hypoxia-Inducible Factor Biology. Cell. Metab..

[19] Li H., Jia Y., Wang Y. (2019). Targeting HIF-1α Signaling Pathway for Gastric Cancer Treatment. Pharmazie.

[20] Schanza L.M., Seles M., Stotz M., Fosselteder J., Hutterer G.C., Pichler M., Stiegelbauer V. (2017). MicroRNAs Associated with Von Hippel-Lindau Pathway in Renal Cell Carcinoma: A Comprehensive Review. Int. J. Mol. Sci..

[21] Ling H., Vincent K., Pichler M., Fodde R., Berindan-Neagoe I., Slack F.J., Calin G.A. (2015). Junk DNA and the Long Non-Coding RNA Twist in Cancer Genetics. Oncogene.

[22] Pichler M., Rodriguez-Aguayo C., Nam S.Y., Dragomir M.P., Bayraktar R., Anfossi S., Knutsen E., Ivan C., Fuentes-Mattei E., Lee S.K. (2020). Therapeutic Potential of FLANC, a Novel Primate-Specific Long Non-Coding RNA in Colorectal Cancer. Gut.

[23] Fosselteder J., Calin G.A., Pichler M. (2018). Long Non-Coding RNA CCAT2 as a Therapeutic Target in Colorectal Cancer. Expert Opin. Ther. Targets.

[24] Cerk S., Schwarzenbacher D., Adiprasito J.B., Stotz M., Hutterer G.C., Gerger A., Ling H., Calin G.A., Pichler M. (2016). Current Status of Long Non-Coding RNAs in Human Breast Cancer. Int. J. Mol. Sci..

[25] Seles M., Hutterer G.C., Kiesslich T., Pummer K., Berindan-Neagoe I., Perakis S., Schwarzenbacher D., Stotz M., Gerger A., Pichler M. (2016). Current Insights into Long Non-Coding RNAs in Renal Cell Carcinoma. Int. J. Mol. Sci..

[26] Smolle M.A., Pichler M. (2018). The Role of Long Non-Coding RNAs in Osteosarcoma. Noncoding RNA.

[27] Barth D.A., Slaby O., Klec C., Juracek J., Drula R., Calin G.A., Pichler M. (2019). Current Concepts of Non-Coding RNAs in the Pathogenesis of Non-Clear Cell Renal Cell Carcinoma. Cancers (Basel).

[28] Ma L., Bajic V.B., Zhang Z. (2013). On the Classification of Long Non-Coding RNAs. RNA Biol..

[29] Wang K., Chang H. (2011). Molecular Mechanisms of Long Noncoding RNAs. Mol. Cell.

[30] Zhang S., Wang W., Liu G., Xie S., Li Q., Li Y., Lin Z. (2017). Long Non-Coding RNA HOTTIP Promotes Hypoxia-Induced Epithelial-Mesenchymal Transition of Malignant Glioma by Regulating the miR-101/ZEB1 Axis. Biomed. Pharm..

[31] Zhang J., Jin H.Y., Wu Y., Zheng Z.C., Guo S., Wang Y., Yang D., Meng X.Y., Xu X., Zhao Y. (2019). Hypoxia-Induced LncRNA PCGEM1 Promotes Invasion and Metastasis of Gastric Cancer through Regulating SNAI1. Clin. Transl. Oncol..

[32] Liu L., Zhao X., Zou H., Bai R., Yang K., Tian Z. (2016). Hypoxia Promotes Gastric Cancer Malignancy Partly through the HIF-1alpha Dependent Transcriptional Activation of the Long Non-Coding RNA GAPLINC. Front. Physiol..

[33] Wei X., Wang C., Ma C., Sun W., Li H., Cai Z. (2016). Long Noncoding RNA ANRIL is Activated by Hypoxia-Inducible Factor-1alpha and Promotes Osteosarcoma Cell Invasion and Suppresses Cell Apoptosis upon Hypoxia. Cancer Cell. Int..

[34] Choudhry H., Harris A.L., McIntyre A. (2016). The Tumour Hypoxia Induced Non-Coding Transcriptome. Mol. Asp. Med..

[35] Wang X., Wang Y., Li L., Xue X., Xie H., Shi H., Hu Y. (2020). A lncRNA Coordinates with Ezh2 to Inhibit HIF-1alpha Transcription and Suppress Cancer Cell Adaption to Hypoxia. Oncogene.

[36] Wang X., Li L., Zhao K., Lin Q., Li H., Xue X., Ge W., He H., Liu D., Xie H. (2020). A Novel LncRNA HITT Forms a Regulatory Loop with HIF-1alpha to Modulate Angiogenesis and Tumor Growth. Cell Death Differ..

[37] Xiang S., Gu H., Jin L., Thorne R.F., Zhang X.D., Wu M. (2018). LncRNA IDH1-AS1 Links the Functions of C-Myc and HIF1alpha Via IDH1 to Regulate the Warburg Effect. Proc. Natl. Acad. Sci. USA.

[38] Shih J.W., Chiang W.F., Wu A.T.H., Wu M.H., Wang L.Y., Yu Y.L., Hung Y.W., Wang W.C., Chu C.Y., Hung C.L. (2017). Long Noncoding RNA LncHIFCAR/MIR31HG is a HIF-1alpha Co-Activator Driving Oral Cancer Progression. Nat. Commun..

[39] Liu D., Li H. (2019). Long Non-Coding RNA GEHT1 Promoted the Proliferation of Ovarian Cancer Cells Via Modulating the Protein Stability of HIF1alpha. Biosci. Rep..

[40] Yang F., Zhang H., Mei Y., Wu M. (2014). Reciprocal Regulation of HIF-1alpha and lincRNA-p21 Modulates the Warburg Effect. Mol. Cell.

[41] Wang Y., Chen W., Lian J., Zhang H., Yu B., Zhang M., Wei F., Wu J., Jiang J., Jia Y. (2020). The lncRNA PVT1 Regulates Nasopharyngeal Carcinoma Cell Proliferation Via Activating the KAT2A Acetyltransferase and Stabilizing HIF-1alpha. Cell Death Differ..

[42] Wang C., Han C., Zhang Y., Liu F. (2018). LncRNA PVT1 Regulate Expression of HIF1alpha Via Functioning as ceRNA for miR199a5p in Nonsmall Cell Lung Cancer Under Hypoxia. Mol. Med. Rep..

[43] Huang T., Liu H.W., Chen J.Q., Wang S.H., Hao L.Q., Liu M., Wang B. (2017). The Long Noncoding RNA PVT1 Functions as a Competing Endogenous RNA by Sponging miR-186 in Gastric Cancer. Biomed. Pharmacother..

[44] Zhao B., Liu K., Cai L. (2019). LINK-A lncRNA Functions in the Metastasis of Osteosarcoma by Upregulating HIF1alpha. Oncol. Lett..

[45] Zhang H., Yao B., Tang S., Chen Y. (2019). LINK-A Long Non-Coding RNA (lncRNA) Participates in Metastasis of Ovarian Carcinoma and Upregulates Hypoxia-Inducible Factor 1 (HIF1alpha). Med. Sci. Monit..

[46] Lin A., Li C., Xing Z., Hu Q., Liang K., Han L., Wang C., Hawke D.H., Wang S., Zhang Y. (2016). The LINK-A lncRNA Activates Normoxic HIF1alpha Signalling in Triple-Negative Breast Cancer. Nat. Cell Biol..

[47] Chen F., Chen J., Yang L., Liu J., Zhang X., Zhang Y., Tu Q., Yin D., Lin D., Wong P.P. (2019). Extracellular Vesicle-Packaged HIF-1alpha-Stabilizing lncRNA from Tumour-Associated Macrophages Regulates Aerobic Glycolysis of Breast Cancer Cells. Nat. Cell Biol..

[48] Zhu Y., Tong Y., Wu J., Liu Y., Zhao M. (2019). Knockdown of LncRNA GHET1 Suppresses Prostate Cancer Cell Proliferation by Inhibiting HIF-1alpha/Notch-1 Signaling Pathway Via KLF2. Biofactors.

[49] Wang R., Ma Z., Feng L., Yang Y., Tan C., Shi Q., Lian M., He S., Ma H., Fang J. (2018). LncRNA MIR31HG Targets HIF1A and P21 to Facilitate Head and Neck Cancer Cell Proliferation and Tumorigenesis by Promoting Cell-Cycle Progression. Mol. Cancer.

[50] Wen X., Liu X., Mao Y.P., Yang X.J., Wang Y.Q., Zhang P.P., Lei Y., Hong X.H., He Q.M., Ma J. (2018). Long Non-Coding RNA DANCR Stabilizes HIF-1alpha and Promotes Metastasis by Interacting with NF90/NF45 Complex in Nasopharyngeal Carcinoma. Theranostics.

[51] Jin Y., Xie H., Duan L., Zhao D., Ding J., Jiang G. (2019). Long Non-Coding RNA CASC9 and HIF-1α Form A Positive Feedback Loop to Facilitate Cell Proliferation and Metastasis in Lung Cancer. OncoTargets Ther..

[52] Su X., Li G., Liu W. (2017). The Long Noncoding RNA Cancer Susceptibility Candidate 9 Promotes Nasopharyngeal Carcinogenesis Via Stabilizing HIF1alpha. DNA Cell Biol..

[53] Luo F., Liu X., Ling M., Lu L., Shi L., Lu X., Li J., Zhang A., Liu Q. (2016). The lncRNA MALAT1, Acting through HIF-1alpha Stabilization, Enhances Arsenite-Induced Glycolysis in Human Hepatic L-02 Cells. Biochim. Biophys. Acta.

[54] Zeng Z., Xu F.Y., Zheng H., Cheng P., Chen Q.Y., Ye Z., Zhong J.X., Deng S.J., Liu M.L., Huang K. (2019). LncRNA-MTA2TR Functions as a Promoter in Pancreatic Cancer Via Driving Deacetylation-Dependent Accumulation of HIF-1alpha. Theranostics.

[55] Lin J., Cao S., Wang Y., Hu Y., Liu H., Li J., Chen J., Li P., Liu J., Wang Q. (2018). Long Non-Coding RNA UBE2CP3 Enhances HCC Cell Secretion of VEGFA and Promotes Angiogenesis by Activating ERK1/2/HIF-1alpha/VEGFA Signalling in Hepatocellular Carcinoma. J. Exp. Clin. Cancer Res..

[56] Zhang T., Wang F., Liao Y., Yuan L., Zhang B. (2019). LncRNA AWPPH Promotes the Invasion and Migration of Glioma Cells through the Upregulation of HIF1alpha. Oncol. Lett..

[57] Yang F., Huo X.S., Yuan S.X., Zhang L., Zhou W.P., Wang F., Sun S.H. (2013). Repression of the Long Noncoding RNA-LET by Histone Deacetylase 3 Contributes to Hypoxia-Mediated Metastasis. Mol. Cell.

[58] Sun Y.W., Chen Y.F., Li J., Huo Y.M., Liu D.J., Hua R., Zhang J.F., Liu W., Yang J.Y., Fu X.L. (2014). A Novel Long Non-Coding RNA ENST00000480739 Suppresses Tumour Cell Invasion by Regulating OS-9 and HIF-1alpha in Pancreatic Ductal Adenocarcinoma. Br. J. Cancer.

[59] Zhang W., Yuan W., Song J., Wang S., Gu X. (2018). LncRNA CPS1-IT1 Suppresses EMT and Metastasis of Colorectal Cancer by Inhibiting Hypoxia-Induced Autophagy through Inactivation of HIF-1alpha. Biochimie.

[60] Wang T.H., Wu C.H., Yeh C.T., Su S.C., Hsia S.M., Liang K.H., Chen C.C., Hsueh C., Chen C.Y. (2017). Melatonin Suppresses Hepatocellular Carcinoma Progression Via lncRNA-CPS1-IT-Mediated HIF-1alpha Inactivation. Oncotarget.

[61] Wang T.H., Yu C.C., Lin Y.S., Chen T.C., Yeh C.T., Liang K.H., Shieh T.M., Chen C.Y., Hsueh C. (2016). Long Noncoding RNA CPS1-IT1 Suppresses the Metastasis of Hepatocellular Carcinoma by Regulating HIF-1alpha Activity and Inhibiting Epithelial-Mesenchymal Transition. Oncotarget.

[62] Zhou C., Huang C., Wang J., Huang H., Li J., Xie Q., Liu Y., Zhu J., Li Y., Zhang D. (2017). LncRNA MEG3 Downregulation Mediated by DNMT3b Contributes to Nickel Malignant Transformation of Human Bronchial Epithelial Cells Via Modulating PHLPP1 Transcription and HIF-1α Translation. Oncogene.

[63] Choi Y.J., Kim I., Lee J.E., Park J.W. (2019). PIN1 Transcript Variant 2 Acts as a Long Non-Coding RNA that Controls the HIF-1-Driven Hypoxic Response. Sci. Rep..

[64] Hamilton M.J., Young M., Jang K., Sauer S., Neang V.E., King A.T., Girke T., Martinez E. (2020). HOTAIRM1 lncRNA is Downregulated in Clear Cell Renal Cell Carcinoma and Inhibits the Hypoxia Pathway. Cancer Lett..

[65] Zhao D., Wang S., Chu X., Han D. (2019). LncRNA HIF2PUT Inhibited Osteosarcoma Stem Cells Proliferation, Migration and Invasion by Regulating HIF2 Expression. Artif. Cells Nanomed. Biotechnol..

[66] Yao J., Li J., Geng P., Li Y., Chen H., Zhu Y. (2015). Knockdown of a HIF-2alpha Promoter Upstream Long Noncoding RNA Impairs Colorectal Cancer Stem Cell Properties in Vitro through HIF-2alpha Downregulation. OncoTargets Ther..

[67] Wang Y., Yao J., Meng H., Yu Z., Wang Z., Yuan X., Chen H., Wang A. (2015). A Novel Long Non-Coding RNA, Hypoxia-Inducible Factor-2alpha Promoter Upstream Transcript, Functions as an Inhibitor of Osteosarcoma Stem Cells in Vitro. Mol. Med. Rep..

[68] Zhai W., Sun Y., Jiang M., Wang M., Gasiewicz T.A., Zheng J., Chang C. (2016). Differential Regulation of LncRNA-SARCC Suppresses VHL-Mutant RCC Cell Proliferation Yet Promotes VHL-Normal RCC Cell Proliferation Via Modulating Androgen Receptor/HIF-2alpha/C-MYC Axis Under Hypoxia. Oncogene.

[69] Luo F., Sun B., Li H., Xu Y., Liu Y., Liu X., Lu L., Li J., Wang Q., Wei S. (2016). A MALAT1/HIF-A Feedback Loop Contributes to Arsenite Carcinogenesis. Oncotarget.

[70] Derderian C., Orunmuyi A.T., Olapade-Olaopa E.O., Ogunwobi O.O. (2019). PVT1 Signaling is a Mediator of Cancer Progression. Front. Oncol..

[71] Pan X., Zheng G., Gao C. (2018). LncRNA PVT1: A Novel Therapeutic Target for Cancers. Clin. Lab..

[72] Wu F., Gao H., Liu K., Gao B., Ren H., Li Z., Liu F. (2019). The lncRNA ZEB2-AS1 is Upregulated in Gastric Cancer and Affects Cell Proliferation and Invasion Via miR-143-5p/HIF-1α Axis. OncoTargets Ther..

[73] Yang L.G., Cao M.Z., Zhang J., Li X.Y., Sun Q.L. (2020). LncRNA XIST Modulates HIF-1A/AXL Signaling Pathway by Inhibiting miR-93-5p in Colorectal Cancer. Mol. Genet. Genom. Med..

[74] Yu X., Hu L., Li S., Shen J., Wang D., Xu R., Yang H. (2019). Long Non-Coding RNA Taurine Upregulated Gene 1 Promotes Osteosarcoma Cell Metastasis by Mediating HIF-1alpha Via miR-143-5p. Cell. Death Dis..

[75] Peng X., Yan J., Cheng F. (2020). LncRNA TMPO-AS1 Up-Regulates the Expression of HIF-1alpha and Promotes the Malignant Phenotypes of Retinoblastoma Cells Via Sponging miR-199a-5p. Pathol. Res. Pract..

[76] Du F., Guo T., Cao C. (2019). Silencing of Long Noncoding RNA SNHG6 Inhibits Esophageal Squamous Cell Carcinoma Progression Via miR-186-5p/HIF1alpha Axis. Dig. Dis. Sci..

[77] Takahashi K., Yan I.K., Haga H., Patel T. (2014). Modulation of Hypoxia-Signaling Pathways by Extracellular Linc-RoR. J. Cell. Sci..

[78] Cai Q., Wang Z., Wang S., Weng M., Zhou D., Li C., Wang J., Chen E., Quan Z. (2017). Long Non-Coding RNA LINC00152 Promotes Gallbladder Cancer Metastasis and Epithelial-Mesenchymal Transition by Regulating HIF-1alpha Via miR-138. Open Biol..

[79] Ou Z., Zhang M., Ji L., Luo Z., Han T., Lu Y., Li Y. (2019). Long Noncoding RNA FEZF1-AS1 Predicts Poor Prognosis and Modulates Pancreatic Cancer Cell Proliferation and Invasion through miR-142/HIF-1α and miR-133a/EGFR upon Hypoxia/Normoxia. J. Cell. Physiol..

[80] Yang B., Jia L., Ren H., Jin C., Ren Q., Zhang H., Hu D., Zhang H., Hu L., Xie T. (2020). LncRNA DLX6-AS1 Increases the Expression of HIF-1alpha and Promotes the Malignant Phenotypes of Nasopharyngeal Carcinoma Cells Via Targeting MiR-199a-5p. Mol. Genet. Genom. Med..

[81] Wang Y., Huang Y., Liu H., Su D., Luo F., Zhou F. (2019). Long Noncoding RNA CDKN2B-AS1 Interacts with miR-411-3p to Regulate Ovarian Cancer in Vitro and in Vivo through HIF-1a/VEGF/P38 Pathway. Biochem. Biophys. Res. Commun..

[82] Li X., Wu Y., Liu A., Tang X. (2016). Long Non-Coding RNA UCA1 Enhances Tamoxifen Resistance in Breast Cancer Cells through a miR-18a-HIF1alpha Feedback Regulatory Loop. Tumour Biol..

[83] Guo X., Lee S., Cao P. (2019). The Inhibitive Effect of Sh-HIF1A-AS2 on the Proliferation, Invasion, and Pathological Damage of Breast Cancer Via Targeting miR-548c-3p through Regulating HIF-1alpha/VEGF Pathway in Vitro and Vivo. OncoTargets Ther..

[84] Hong Q., Li O., Zheng W., Xiao W.Z., Zhang L., Wu D., Cai G.Y., He J.C., Chen X.M. (2017). LncRNA HOTAIR Regulates HIF-1alpha/AXL Signaling through Inhibition of miR-217 in Renal Cell Carcinoma. Cell. Death Dis..

[85] Zhou C., Ye L., Jiang C., Bai J., Chi Y., Zhang H. (2015). Long Noncoding RNA HOTAIR, a Hypoxia-Inducible Factor-1α activated Driver of Malignancy, Enhances Hypoxic Cancer Cell Proliferation, Migration, and Invasion in Non-Small Cell Lung Cancer. Tumor Biol..

[86] Li N., Meng D.D., Gao L., Xu Y., Liu P.J., Tian Y.W., Yi Z.Y., Zhang Y., Tie X.J., Xu Z.Q. (2018). Overexpression of HOTAIR Leads to Radioresistance of Human Cervical Cancer Via Promoting HIF-1α expression. Radiat. Oncol..

[87] Hu M., Fu Q., Jing C., Zhang X., Qin T., Pan Y. (2020). LncRNA HOTAIR Knockdown Inhibits Glycolysis by Regulating miR-130a-3p/HIF1A in Hepatocellular Carcinoma Under Hypoxia. Biomed. Pharm..

[88] Peng F., Wang J., Fan W., Meng Y., Li M., Li T., Cui B., Wang H., Zhao Y., An F. (2018). Glycolysis Gatekeeper PDK1 Reprograms Breast Cancer Stem Cells Under Hypoxia. Oncogene.

[89] Zhu H., Jin Y., Lyu X., Fan L., Wu F. (2019). Long Noncoding RNA H19 Regulates HIF-1α/AXL Signaling through Inhibiting miR-20b-5p in Endometrial Cancer. Cell Cycle.

[90] Corrado C., Costa V., Giavaresi G., Calabrese A., Conigliaro A., Alessandro R. (2019). Long Non Coding RNA H19: A New Player in Hypoxia-Induced Multiple Myeloma Cell Dissemination. Int. J. Mol. Sci..

[91] Tan H., Zhao L. (2019). lncRNA Nuclear-Enriched Abundant Transcript 1 Promotes Cell Proliferation and Invasion by Targeting miR-186-5p/HIF-1alpha in Osteosarcoma. J. Cell. Biochem..

[92] Ghafouri-Fard S., Esmaeili M., Taheri M. (2020). H19 lncRNA: Roles in Tumorigenesis. Biomed. Pharm..

[93] Yoshimura H., Matsuda Y., Yamamoto M., Kamiya S., Ishiwata T. (2018). Expression and Role of Long Non-Coding RNA H19 in Carcinogenesis. Front. Biosci. (Landmark Ed.).

[94] Matouk I.J., Mezan S., Mizrahi A., Ohana P., Abu-Lail R., Fellig Y., Degroot N., Galun E., Hochberg A. (2010). The Oncofetal H19 RNA Connection: Hypoxia, p53 and Cancer. Biochim. Biophys. Acta.

[95] Wu W., Hu Q., Nie E., Yu T., Wu Y., Zhi T., Jiang K., Shen F., Wang Y., Zhang J. (2017). Hypoxia Induces H19 Expression through Direct and Indirect Hif-1α Activity, Promoting Oncogenic Effects in Glioblastoma. Sci. Rep..

[96] Papandreou I., Cairns R.A., Fontana L., Lim A.L., Denko N.C. (2006). HIF-1 Mediates Adaptation to Hypoxia by Actively Downregulating Mitochondrial Oxygen Consumption. Cell. Metab..

[97] Lo Dico A., Costa V., Martelli C., Diceglie C., Rajata F., Rizzo A., Mancone C., Tripodi M., Ottobrini L., Alessandro R. (2016). MiR675-5p Acts on HIF-1alpha to Sustain Hypoxic Responses: A New Therapeutic Strategy for Glioma. Theranostics.

[98] Qu X., Alsager S., Zhuo Y., Shan B. (2019). HOX Transcript Antisense RNA (HOTAIR) in Cancer. Cancer Lett..

[99] Li T., Xiao Y., Huang T. (2018). HIF1alphainduced Upregulation of lncRNA UCA1 Promotes Cell Growth in Osteosarcoma by Inactivating the PTEN/AKT Signaling Pathway. Oncol. Rep..

[100] Xue M., Li X., Li Z., Chen W. (2014). Urothelial Carcinoma Associated 1 is a Hypoxia-Inducible Factor-1alpha-Targeted Long Noncoding RNA that Enhances Hypoxic Bladder Cancer Cell Proliferation, Migration, and Invasion. Tumour Biol..

[101] Yang J., Li L., Hong S., Zhou Z., Fan W. (2019). LINK-A lncRNA Activates HIF1alpha Signaling and Inhibits Podocyte Cell Apoptosis in Diabetic Nephropathy. Exp. Ther. Med..

[102] Ye Y., Peng Y., Li Y., Liu C., Xu Y., Li W. (2019). Effect of lincRNA-p21 Targeting HIF-1alpha on Biological Functions of Liver Cancer Cells. Oncol. Lett..

[103] Shen Y., Liu Y., Sun T., Yang W. (2017). LincRNA-p21 Knockdown Enhances Radiosensitivity of Hypoxic Tumor Cells by Reducing Autophagy through HIF-1/Akt/mTOR/P70S6K Pathway. Exp. Cell Res..

[104] Maolake A., Izumi K., Shigehara K., Natsagdorj A., Iwamoto H., Kadomoto S., Takezawa Y., Machioka K., Narimoto K., Namiki M. (2017). Tumor-Associated Macrophages Promote Prostate Cancer Migration through Activation of the CCL22-CCR4 Axis. Oncotarget.

[105] De Palma M., Biziato D., Petrova T.V. (2017). Microenvironmental Regulation of Tumour Angiogenesis. Nat. Rev. Cancer.

[106] Goswami S., Sahai E., Wyckoff J.B., Cammer M., Cox D., Pixley F.J., Stanley E.R., Segall J.E., Condeelis J.S. (2005). Macrophages Promote the Invasion of Breast Carcinoma Cells Via a Colony-Stimulating Factor-1/Epidermal Growth Factor Paracrine Loop. Cancer Res..

[107] Wang H.G., Cao B., Zhang L.X., Song N., Li H., Zhao W.Z., Li Y.S., Ma S.M., Yin D.J. (2017). KLF2 Inhibits Cell Growth Via Regulating HIF-1alpha/Notch-1 Signal Pathway in Human Colorectal Cancer HCT116 Cells. Oncol. Rep..

[108] Jin L., He Y., Tang S., Huang S. (2018). LncRNA GHET1 Predicts Poor Prognosis in Hepatocellular Carcinoma and Promotes Cell Proliferation by Silencing KLF2. J. Cell. Physiol..

[109] Augoff K., McCue B., Plow E.F., Sossey-Alaoui K. (2012). miR-31 and its Host Gene lncRNA LOC554202 are Regulated by Promoter Hypermethylation in Triple-Negative Breast Cancer. Mol. Cancer.

[110] Nie F.Q., Ma S., Xie M., Liu Y.W., De W., Liu X.H. (2016). Decreased Long Noncoding RNA MIR31HG is Correlated with Poor Prognosis and Contributes to Cell Proliferation in Gastric Cancer. Tumour Biol..

[111] Wang B., Jiang H., Wang L., Chen X., Wu K., Zhang S., Ma S., Xia B. (2017). Increased MIR31HG lncRNA Expression Increases Gefitinib Resistance in Non-Small Cell Lung Cancer Cell Lines through the EGFR/PI3K/AKT Signaling Pathway. Oncol. Lett..

[112] Yang H., Liu P., Zhang J., Peng X., Lu Z., Yu S., Meng Y., Tong W.M., Chen J. (2016). Long Noncoding RNA MIR31HG Exhibits Oncogenic Property in Pancreatic Ductal Adenocarcinoma and is Negatively Regulated by miR-193b. Oncogene.

[113] Dandan W., Jianliang C., Haiyan H., Hang M., Xuedong L. (2019). Long Noncoding RNA MIR31HG is Activated by SP1 and Promotes Cell Migration and Invasion by Sponging miR-214 in NSCLC. Gene.

[114] Zheng S., Zhang X., Wang X., Li J. (2019). MIR31HG Promotes Cell Proliferation and Invasion by Activating the Wnt/Beta-Catenin Signaling Pathway in Non-Small Cell Lung Cancer. Oncol. Lett..

[115] Castella S., Bernard R., Corno M., Fradin A., Larcher J.C. (2015). Ilf3 and NF90 Functions in RNA Biology. Wiley Interdiscip. Rev. RNA.

[116] Schmidt T., Friedrich S., Golbik R.P., Behrens S.E. (2017). NF90-NF45 is a Selective RNA Chaperone that Rearranges Viral and Cellular Riboswitches: Biochemical Analysis of a Virus Host Factor Activity. Nucleic Acids Res..

[117] Sun Y., Ma L. (2019). New Insights into Long Non-Coding RNA MALAT1 in Cancer and Metastasis. Cancers (Basel).

[118] Hu L., Tang J., Huang X., Zhang T., Feng X. (2018). Hypoxia Exposure Upregulates MALAT-1 and Regulates the Transcriptional Activity of PTB-Associated Splicing Factor in A549 Lung Adenocarcinoma Cells. Oncol. Lett..

[119] Ikeda S., Kitadate A., Abe F., Takahashi N., Tagawa H. (2018). Hypoxia-Inducible KDM3A Addiction in Multiple Myeloma. Blood Adv..

[120] Zhu S., Deng S., He C., Liu M., Chen H., Zeng Z., Zhong J., Ye Z., Deng S., Wu H. (2018). Reciprocal Loop of Hypoxia-Inducible Factor-1α (HIF-1α) and Metastasis-Associated Protein 2 (MTA2) Contributes to the Progression of Pancreatic Carcinoma by Suppressing E-Cadherin Transcription. J. Pathol..

[121] Liu A.N., Qu H.J., Gong W.J., Xiang J.Y., Yang M.M., Zhang W. (2019). LncRNA AWPPH and miRNA-21 Regulates Cancer Cell Proliferation and Chemosensitivity in Triple-Negative Breast Cancer by Interacting with each Other. J. Cell. Biochem..

[122] Huo Y., Li A., Wang Z. (2019). LncRNA AWPPH Participates in the Metastasis of Non-Small Cell Lung Cancer by Upregulating TGF-Beta1 Expression. Oncol. Lett..

[123] Li C., Wang F., Wei B., Wang L., Kong D. (2019). LncRNA AWPPH Promotes Osteosarcoma Progression Via Activation of Wnt/Beta-Catenin Pathway through Modulating miR-93-3p/FZD7 Axis. Biochem. Biophys. Res. Commun..

[124] Weidle U.H., Birzele F., Kollmorgen G., Ruger R. (2017). Long Non-Coding RNAs and their Role in Metastasis. Cancer. Genom. Proteom..

[125] Zhuang J., Shen L., Yang L., Huang X., Lu Q., Cui Y., Zheng X., Zhao X., Zhang D., Huang R. (2017). TGFbeta1 Promotes Gemcitabine Resistance through Regulating the LncRNA-LET/NF90/miR-145 Signaling Axis in Bladder Cancer. Theranostics.

[126] Mao Z., Li H., Du B., Cui K., Xing Y., Zhao X., Zai S. (2017). LncRNA DANCR Promotes Migration and Invasion through Suppression of lncRNA-LET in Gastric Cancer Cells. Biosci. Rep..

[127] Piunti A., Shilatifard A. (2016). Epigenetic Balance of Gene Expression by Polycomb and COMPASS Families. Science.

[128] Newton A.C., Trotman L.C. (2014). Turning off AKT: PHLPP as a Drug Target. Annu. Rev. Pharm. Toxicol..

[129] Dang C.V. (2012). MYC on the Path to Cancer. Cell.

[130] MacKenzie E.D., Selak M.A., Tennant D.A., Payne L.J., Crosby S., Frederiksen C.M., Watson D.G., Gottlieb E. (2007). Cell-Permeating Alpha-Ketoglutarate Derivatives Alleviate Pseudohypoxia in Succinate Dehydrogenase-Deficient Cells. Mol. Cell. Biol..

[131] Lonati E., Brambilla A., Milani C., Masserini M., Palestini P., Bulbarelli A. (2014). Pin1, a New Player in the Fate of HIF-1alpha Degradation: An Hypothetical Mechanism Inside Vascular Damage as Alzheimer’s Disease Risk Factor. Front. Cell. Neurosci..

[132] Jalouli M., Dery M.A., Lafleur V.N., Lamalice L., Zhou X.Z., Lu K.P., Richard D.E. (2014). The Prolyl Isomerase Pin1 Regulates Hypoxia-Inducible Transcription Factor (HIF) Activity. Cell. Signal..

[133] Han H.J., Kwon N., Choi M.A., Jung K.O., Piao J.Y., Ngo H.K., Kim S.J., Kim D.H., Chung J.K., Cha Y.N. (2016). Peptidyl Prolyl Isomerase PIN1 Directly Binds to and Stabilizes Hypoxia-Inducible Factor-1alpha. PLoS ONE.

[134] Li W., He X., Xue R., Zhang Y., Zhang X., Lu J., Zhang Z., Xue L. (2016). Combined Over-Expression of the Hypoxia-Inducible Factor 2alpha Gene and its Long Non-Coding RNA Predicts Unfavorable Prognosis of Patients with Osteosarcoma. Pathol. Res. Pract..

[135] Chen Y., Sun Y., Rao Q., Xu H., Li L., Chang C. (2015). Androgen Receptor (AR) Suppresses miRNA-145 to Promote Renal Cell Carcinoma (RCC) Progression Independent of VHL Status. Oncotarget.

[136] He D., Li L., Zhu G., Liang L., Guan Z., Chang L., Chen Y., Yeh S., Chang C. (2014). ASC-J9 Suppresses Renal Cell Carcinoma Progression by Targeting an Androgen Receptor-Dependent HIF2alpha/VEGF Signaling Pathway. Cancer Res..

[137] Yuan P., Cao W., Zang Q., Li G., Guo X., Fan J. (2016). The HIF-2alpha-MALAT1-miR-216b Axis Regulates Multi-Drug Resistance of Hepatocellular Carcinoma Cells Via Modulating Autophagy. Biochem. Biophys. Res. Commun..

[138] Klec C., Prinz F., Pichler M. (2019). Involvement of the Long Noncoding RNA NEAT1 in Carcinogenesis. Mol. Oncol..

[139] Dong P., Xiong Y., Yue J., Hanley S.J.B., Kobayashi N., Todo Y., Watari H. (2018). Long Non-Coding RNA NEAT1: A Novel Target for Diagnosis and Therapy in Human Tumors. Front. Genet..

[140] Zheng X., Zhang Y., Liu Y., Fang L., Li L., Sun J., Pan Z., Xin W., Huang P. (2018). HIF-2α Activated lncRNA NEAT1 Promotes Hepatocellular Carcinoma Cell Invasion and Metastasis by Affecting the Epithelial-Mesenchymal Transition. J. Cell. Biochem..

[141] Choudhry H., Albukhari A., Morotti M., Haider S., Moralli D., Smythies J., Schodel J., Green C.M., Camps C., Buffa F. (2015). Tumor Hypoxia Induces Nuclear Paraspeckle Formation through HIF-2alpha Dependent Transcriptional Activation of NEAT1 Leading to Cancer Cell Survival. Oncogene.

[142] Kong X., Zhao Y., Li X., Tao Z., Hou M., Ma H. (2019). Overexpression of HIF-2alpha-Dependent NEAT1 Promotes the Progression of Non-Small Cell Lung Cancer through miR-101-3p/SOX9/Wnt/Beta-Catenin Signal Pathway. Cell. Physiol. Biochem..

[143] Zhang P., Cao L., Zhou R., Yang X., Wu M. (2019). The lncRNA Neat1 Promotes Activation of Inflammasomes in Macrophages. Nat. Commun..

